# Prevalence and predisposing factors for fatigue in patients with chronic renal disease undergoing hemodialysis: a cross-sectional study

**DOI:** 10.1590/1516-3180.2022.0127.R1.01122022

**Published:** 2023-04-07

**Authors:** Ricardo Eugenio Mariani Burdelis, Felipe José Silva Melo Cruz

**Affiliations:** I MSc. Physician, Faculdade de Medicina do ABC (FMABC), Santo André (SP), Brazil.; II PhD. Physician, Faculdade de Medicina do ABC (FMABC), Santo André (SP), Brazil. Physician, Department of Oncology, Núcleo de Ensino e Pesquisa da Rede São Camilo, São Paulo (SP), Brazil

**Keywords:** Renal replacement therapy, Fatigue, Quality of life, Chronic kidney disease, Inflammatory markers, Dialysis patients

## Abstract

**BACKGROUND:**

Patients with chronic renal disease and undergoing hemodialysis are at a high risk for developing several complications. Fatigue is a common, troubling symptom that affects such patients and can contribute to unfavorable outcomes and high mortality.

**OBJECTIVE:**

This cross-sectional study aimed to evaluate the prevalence of fatigue in Brazilian patients with chronic kidney disease undergoing hemodialysis and determine the predisposing factors for fatigue.

**DESIGN AND SETTING:**

An observational, cross-sectional, descriptive study was conducted in two renal replacement therapy centers in the Greater ABC region of São Paulo.

**METHODS:**

This study included 95 patients undergoing dialysis who were consecutively treated at two Brazilian renal replacement therapy centers between September 2019 and February 2020. The Chalder questionnaire was used to evaluate fatigue. Clinical, sociodemographic, and laboratory data of the patients were recorded, and the Short Form 36 Health Survey, Pittsburgh Sleep Quality Index, and Beck Depression Inventory were administered.

**RESULTS:**

The prevalence of fatigue in patients undergoing hemodialysis was 51.6%. Fatigue was independently associated with lower quality of life in terms of physical and general health. Patients with fatigue had a higher incidence of depression (65.9% vs. 34.1%, P = 0.001) and worse sleep quality (59.1% vs. 49.9%; P = 0.027) than those without fatigue.

**CONCLUSION:**

Prevalence of fatigue is high in patients undergoing hemodialysis and is directly related to physical and general health.

## INTRODUCTION

Patients with chronic renal disease undergoing hemodialysis are at a higher risk of developing several complications, including infection, cardiovascular and bone disease, and metabolic changes.^1,2^ The prevalence of chronic kidney disease is exponentially increasing in Brazil, with 596 patients per million undergoing dialysis and an annual gross mortality rate of 18.2%.^3^

Patients undergoing renal replacement therapy present with varying levels of disease severity that compromise their quality of life and affect their physical and psychological health. Dialysis-induced changes include physical, self-care, and social activity limitations, intense body pain, frequent episodes of fatigue, and poor self-assessment of physical health. Mental changes include psychological distress, emotional problems related to the social impact of treatment, and poor mental health assessment.^4^

A meta-analysis of patients undergoing hemodialysis showed that physical and emotional symptoms were also associated with depressive symptoms.^1^ Furthermore, patients with signs of depression report fatigue.^1^ Thus, it is believed that fatigue and depression or mood disorders may share the same pathogenic pathway.^5^ Sleeping disorders are frequently associated with fatigue as well.^5,6^

The definition of fatigue remains unclear and is often characterized by an increased feeling of weakness, tiredness, and lack of energy. Furthermore, it is described as a physical and mental experience.^7,8^

Several multifaceted and multidimensional factors affect fatigue in patients with chronic renal disease undergoing hemodialysis.^7^ Although it is a prevalent symptom in patients undergoing dialysis, fatigue has been poorly studied in Brazilian patients.

## OBJECTIVE

This cross-sectional study aimed to assess the prevalence of fatigue in patients with chronic kidney disease undergoing renal replacement therapy in the form of hemodialysis at two dialysis centers in the ABC Paulista region. This study also examined the predisposing factors for fatigue in the study population.

## METHODS

This observational, cross-sectional, descriptive study analyzed the prevalence of and predisposing factors for fatigue in patients with chronic kidney disease undergoing hemodialysis. The study was conducted at two renal replacement therapy centers in the Greater ABC region of São Paulo. One of the centers was located at a high-complexity hospital treating patients from the Unified Health System; the other was located in a center treating patients from the private sector.

Patients with stage 5 chronic kidney disease who were undergoing hemodialysis were eligible to participate in the study. The exclusion criteria were as follows: peritoneal dialysis, age < 21 years, dialysis for < 12 months, psychiatric disorders with cognitive deterioration, active infectious or autoimmune disease, liver failure, and metastatic malignant neoplasms.

The study protocol was approved by our institution’s ethics committees for research on humans under the CAAE number 24471419.7.0000.0082 (Decision number: 3.705.408) on November 14, 2019. The study was conducted in accordance with the Declaration of Helsinki. All participants provided written informed consents before any study-related procedures were performed.

The patients’ demographic characteristics (age, sex, race, marital status, education, and income) were collected directly from clinical databases and records. Clinical data, including the cause of chronic kidney disease, existing comorbidities, medications being used, treatment complications, and duration of dialysis, were collected from the patients’ medical records. The patients’ laboratory parameters, including the serum hemoglobin, albumin, urea, parathyroid hormone, ferritin, calcium, phosphorus, and potassium levels and dialysis adequacy were also collected from the system.

Patients were evaluated during a single consultation and instructed to complete the following surveys: Chalder Fatigue Questionnaire, 36-Item Short Form Survey (SF-36), Beck Depression Inventory II (BDI-II), and Pittsburgh Sleep Quality Index (PSQI).

The Chalder Fatigue Scale is a self-administered questionnaire used to measure the extent and severity of fatigue in both clinical and non-clinical epidemiological populations. This scale consists of 11 items which are answered on a 4-point scale ranging from asymptomatic to maximum symptomology (“better than usual,” “no worse than usual,” “worse than usual,” and “much worse than usual”). The total score ranges from 0 to 33 and spans two dimensions: physical and psychological fatigue.^9^

The SF-36 questionnaire consists of the following eight multi-item scales: physical functioning, body pain, mental health, general health, vitality, role limitation due to emotional problems, role limitations due to physical health, and social functioning. The scores range from 0 to 100, where 0 corresponds to bad health and 100 corresponds to good health.^10^

BDI-II is a self-administered scale used worldwide to detect depressive symptoms. This questionnaire consists of 21 statements about depression ranked on an ordinal scale from 0 to 3, resulting in a total score ranging from 0 to 63.^11^

The PSQI is a self-evaluation tool developed by Buysse that assesses sleep quality. It consists of 18 items, and the total score ranges from 0 to 21. A score ≤ 7 indicates good sleep quality; a score > 7 indicates poor sleep quality. This questionnaire has been widely used to measure sleep quality in different groups of patients, including those with kidney and intestinal diseases, asthma, and cancer.^12^

In both institutions, dialysis was performed in three shifts per day; each session lasted for four hours with a half hour in between for reorganizing the rooms. Dialysis sessions started at 06:00 and ended at 19:00. Questionnaires were administered by an investigator and a nurse responsible for each dialysis, who had been duly trained for this study.

### Statistical analysis

Qualitative variables are described using absolute and relative frequencies, whereas, quantitative variables are presented as summary measures (mean, standard deviation, median, minimum, and maximum).^13^

The prevalence of fatigue was analyzed according to each qualitative characteristic, using absolute and relative frequencies. Chi-square or likelihood ratio tests were used to evaluate the association between the characteristics and presence of fatigue. Quantitative characteristics were described in terms of their association with fatigue and compared using Student’s t- or Mann-Whitney U-tests.^13^

Odds ratios (OR) were calculated with unadjusted 95% confidence intervals (CI). A binary logistic regression model was used to identify the presence or absence of fatigue for each of the evaluated characteristics. The model included descriptive sex and age characteristics with a P value < 0.20 and the probability for fatigue. A backward stepwise regression selection method was used, with the input and output criteria of the final model at 5%.^14^

All statistical analyses were performed using the Statistical Package for Social Sciences (SPSS) for Windows (version 20.0; IBM, Armonk, New York, United States). Clinical significance was set at P < 0.05.

## RESULTS

**Figure 1.**
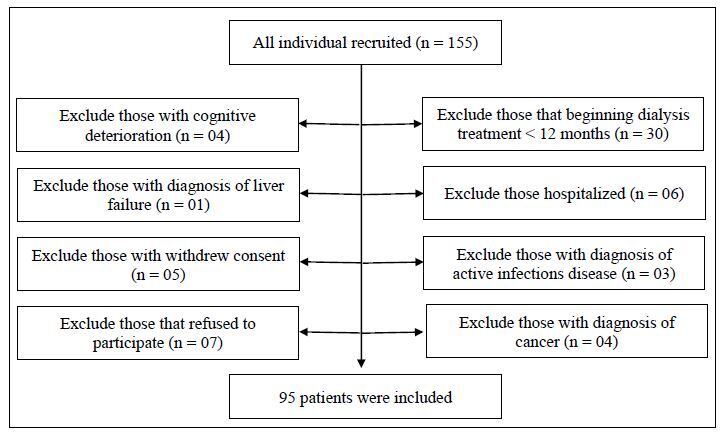
Flow chart depicting the inclusion and exclusion of the study population.

A total of 155 patients were registered at two hemodialysis clinics in the Greater ABC region of São Paulo. Of these, 60 patients were excluded for the following reasons: cognitive deterioration (4), diagnosis of liver failure (1), withdrawal of consent (5), refusal to participate (7), dialysis for <12 months (30), hospitalization (6), diagnosis of an active infection (3), and diagnosis of cancer (4). The remaining eligible 95 patients, who were diagnosed with chronic kidney disease and were undergoing hemodialysis were included in the study (Figure1). After obtaining patient consent, relevant data were extracted from their medical records, including from physician and nursing notes and diagnostic tests. The medical records of the 95 eligible patients were complete and kept updated because these patients underwent monthly routine examinations and medical consultations to obtain information necessary for determining treatment plan.

The demographic characteristics of the participants are presented in Table 1. There were 53 (55.8%) female and 42 (44.2%) male patients with a mean age of 56.4 ± 14.3 years. Most patients had a low educational level and economic status (78.9% were unemployed and only 10.6% had an income four times above the national/regional/local minimum wage). The main reported comorbidities were hypertension (40%) and diabetes (33.7%); most of the patients led a sedentary lifestyle (78.9%).

**Table 1.** Demographic characteristics and clinical variables (n = 95)

Variables	Number of patients (%)
**Sex**	
Male	42 (44.2)
Female	53 (55.8)
**Age, years**	
Mean ± SD	56.4 ± 14.3
Median (min.; max.)	57 (24; 87)
**Marital status**	
Unmarried	29 (30.5)
Married	49 (51.6)
Divorced	8 (8.4)
Widowed	9 (9.5)
**Race**	
White	49 (51.6)
Black	22 (23.2)
Other	24 (25.3)
**Religion**	
Catholic	45 (47.4)
Evangelical	26 (27.4)
Others	24 (25.3)
**Education**	
Illiterate	8 (8.4)
Elementary school	45 (47.4)
High school	32 (33.7)
University	10 (10.5)
**Employment status**	
Not employed	75 (78.9)
Being employed	20 (21.1)
**Household number**	
Mean ± SD	2.89 ± 1.46
Median (min.; max.)	2 (1; 7)
**Family income**	
Up to 2 times minimum wage	46 (48.4)
2–4 times minimum wage	39 (41.1)
4–10 times minimum wage	9 (9.5)
10–20 times minimum wage	1 (1.1)
**Etiology of kidney disease**	
Hypertensive nephrosclerosis	28 (29.5)
Diabetic nephropathy	35 (36.8)
Chronic glomerulonephritis	3 (3.2)
Other	29 (30.5)
**Comorbidity**	
None	13 (13.7)
Diabetes mellitus	32 (33.7)
Hypertension	38 (40)
Other	12 (12.6)
**Regular exercise**	
No	75 (78.9)
Yes	20 (21.1)
**Hospitalization**	
No	38 (40)
Yes	57 (60)
**Time on dialysis, years**	
Mean ± SD	3.94 ± 4.04
Median (min.; max.)	3 (0.3; 24)

SD = standard deviation; min = minimum; max = maximum.

The laboratory examination results of the patients’ samples were predominantly within the normal range expected for the population studied.^15^ There were no significant differences in the laboratory values between patients with or without fatigue.

All the patients in our study had a low quality of life, depressive symptoms (34.7%), and a high prevalence of sleep disorders (69.5%). Among the 95 enrolled patients undergoing hemodialysis, the prevalence of fatigue was 51.6% (Table 2). Although depression was more frequently seen in women in our study, there was no significant difference in the incidence of depression in either sex (P = 0.392) (data not shown).

**Table 2.** Responses to the Quality of life questionnaire

Variable	Description
**Functional capacity**	
Mean ± SD	48.7 ± 32.1
Median (min.; max.)	50 (0; 100)
**Physical health**	
Mean ± SD	33.2 ± 40.2
Median (min.; max.)	25 (0; 100)
**Pain**	
Mean ± SD	57.4 ± 29.8
Median (min.; max.)	51 (0; 100)
**General health**	
Mean ± SD	46.1 ± 20.7
Median (min.; max.)	47 (5; 100)
**Vitality**	
Mean ± SD	52.1 ± 23.3
Median (min.; max.)	50 (5; 100)
**Social aspect**	
Mean ± SD	65.9 ± 27.8
Median (min.; max.)	62.5 (0; 100)
**Emotional aspect**	
Mean ± SD	47.7 ± 42
Median (min.; max.)	33.3 (0; 100)
**Mental health**	
Mean ± SD	64.3 ± 23
Median (min.; max.)	68 (12; 100)
**BDI-II, n (%)**	
Normal	41 (43.2)
Mild disorder	21 (22.1)
Onset of clinical depression	14 (14.7)
Moderate depression	14 (14.7)
Severe depression	4 (4.2)
Extreme depression	1 (1.1)
**PSQI, n (%)**	
Bad	66 (69.5)
Good	29 (30.5)
**Fatigue, n (%)**	
No	46 (48.4)
Yes	49 (51.6)

BDI-II = Beck Depression Inventory II; PSQI = Pittsburg Sleep Quality Index; SD = standard deviation; min = minimum; max = maximum; n = number of observations.

Binary analysis revealed that fatigue was not significantly associated with demographic or clinical characteristics when analyzed in isolation (P > 0.05) (Table 3). However, quality of life domains were significantly lower in patients with fatigue than in those without fatigue (P < 0.05). Additionally, the prevalence of depression (P = 0.001) and poor sleep quality (P = 0.027) were significantly higher among patients with fatigue than in those without fatigue (Table 4).

**Table 3.** Laboratory results of patients based on the presence of fatigue and the bivariate analysis results

Variable	Fatigue		OR	95% CI		P
	No	Yes		Lower	Upper	
**Kt/V**			0.831	0.333	2.074	0.696**
Mean ± SD	1.49 ± 0.5	1.45 ± 0.39				
Median (min.; max.)	1.6 (0.08; 2.68)	1.5 (0.75; 2.8)				
**Kt/V, n (%)**						0.087
Major 1.2	38 (53.5)	33 (46.5)	1.00			
Minor 1.2	8 (33.3)	16 (66.7)	2.30	0.87	6.07	
**Hemoglobin**			0.896	0.737	1.090	0.271**
Mean ± SD	11.6 ± 1.8	11.1 ± 2.4				
Median (min.; max.)	11.6 (5.1; 14.4)	11.3 (2.6; 15.3)				
**Albumin**			1.023	0.344	3.044	0.967**
Mean ± SD	3.99 ± 0.32	3.99 ± 0.42				
Median (min.; max.)	4 (3; 4.7)	4.03 (2.2; 4.98)				
**Urea pre-dialysis**			0.996	0.985	1.008	0.537**
Mean ± SD	156.8 ± 38	152.3 ± 33.5				
Median (min.; max.)	158.5 (94; 264)	149.4 (93.2; 226)				
**Urea post-dialysis**			1.002	0.982	1.023	0.840**
Mean ± SD	51.3 ± 21.7	52.1 ± 18.1				
Median (min.; max.)	48.4 (4.2; 134)	47.6 (26.6; 103)				
**Parathormone**			1.000	0.998	1.002	0.571^¢^
Mean ± SD	299.7 ± 206.2	293.6 ± 267.8				
Median (min.; max.)	257.8 (22.4; 759.4)	212.9 (33.4; 1620.9)				
**Ferritin**			0.999	0.997	1.000	0.110**
Mean ± SD	349.3 ± 307.6	263.3 ± 201.1				
Median (min.; max.)	258.6 (28.7; 1431.6)	200.2 (34.9; 792.8)				
**Calcium**			0.727	0.413	1.280	0.270**
Mean ± SD	8.7 ± 0.7	8.6 ± 0.7				
Median (min.; max.)	8.7 (7; 11)	8.5 (7.3; 10.8)				
**Phosphorus**			0.851	0.665	1.090	0.201**
Mean ± SD	5.38 ± 1.77	4.94 ± 1.6				
Median (min.; max.)	4.85 (1.8; 10.2)	5 (1.6; 9.6)				
**Potassium**			0.899	0.532	1.520	0.695**
Mean ± SD	4.85 ± 0.77	4.79 ± 0.78				
Median (min.; max.)	4.8 (3.5; 6.4)	4.61 (3.1; 7)				

Chi-square test (χ^2^); **Student’s t-test; ^¢^Mann-Whitney test. SD = standard deviation; min = minimum; max = maximum; OR = odds ratio; CI = confidence interval.

**Table 4.** Questionnaire responses according to the presence of fatigue and the bivariate analysis results

Variable	Fatigue		OR	CI (95%)		P
	No	Yes		Lower	Higher	
**Functional capacity**			0.970	0.956	0.985	**< 0.001** ^¢^
Mean ± SD	62.7 ± 30.2	35.5 ± 28.2				
Median (min.; max.)	70 (0; 100)	30 (0; 100)				
**Physical health**			0.976	0.964	0.988	**< 0.001** ^¢^
Mean ± SD	51.1 ± 43.4	16.3 ± 28.2				
Median (min.; max.)	50 (0; 100)	0 (0; 100)				
**Pain**			0.976	0.961	0.991	**0.001** ^¢^
Mean ± SD	67.5 ± 25.8	47.9 ± 30.3				
Median (min.; max.)	62 (0; 100)	41 (0; 100)				
**General health**			0.960	0.937	0.983	**< 0.001** ^¢^
Mean ± SD	54 ± 21.5	38.8 ± 17.1				
Median (min.; max.)	57 (5; 100)	42 (5; 77)				
**Vitality**			0.961	0.940	0.982	**< 0.001** ^¢^
Mean ± SD	61.7 ± 22.6	43.1 ± 20.2				
Median (min.; max.)	62.5 (15; 100)	45 (5; 80)				
**Social aspect**			0.964	0.947	0.982	**< 0.001** ^¢^
Mean ± SD	78.3 ± 23.9	54.3 ± 26.3				
Median (min.; max.)	87.5 (12.5; 100)	50 (0; 100)				
**Emotional aspect**			0.980	0.970	0.991	**< 0.001** ^¢^
Mean ± SD	64.5 ± 41.8	32 ± 36				
Median (min.; max.)	100 (0; 100)	33.3 (0; 100)				
**Mental health**			0.969	0.950	0.989	**0.002** ^¢^
Mean ± SD	72.1 ± 19.8	57.1 ± 23.5				
Median (min.; max.)	76 (24; 100)	60 (12; 100)				
**BDI-II, n (%)**			1.823	1.260	2.637	**0.001** ^¢^
Normal	27 (65.9)	14 (34.1)				
Mild disorder	11 (52.4)	10 (47.6)				
Onset of clinical depression	3 (21.4)	11 (78.6)				
Moderate depression	4 (28.6)	10 (71.4)				
Severe depression	1 (25)	3 (75)				
Extreme depression	0 (0)	1 (100)				
**PSQI, n (%)**						**0.027**
Bad	27 (49.9)	39 (59.1)	1.00			
Good	19 (65.5)	10 (34.5)	0.36	0.15	0.91	

Chi-square test (χ^2^); ^¢^Mann-Whitney test. BDI-II = Beck Depression Inventory II; PSQI = Pittsburgh Sleep Quality Index; SD = standard deviation; min = minimum; max = maximum; OR = odds ratio; CI = confidence interval.

In the final adjusted model, regardless of the other evaluated characteristics, the domains of physical and general health in the joint quality of life significantly influenced the presence of fatigue in patients with chronic kidney disease undergoing hemodialysis (P < 0.05). The probability of being fatigued decreased by 2% for each percentage of increase in the patient’s physical health. The probability of being fatigued decreased by 3% for each additional percentage of increase in the patient’s general health.

## DISCUSSION

Fatigue has a high prevalence among patients with chronic kidney disease worldwide, with several unfavorable outcomes. Fatigue is considered to be an independent risk factor for increased mortality in such patients.^16^ Although studies on this subject are scarce in Brazil, our findings suggest that the observed high prevalence of fatigue is comparable to the current scientific evidence.^17,18^ There was no statistical difference in the presence of fatigue between the sexes; this result is in contrast to previous studies that suggest that fatigue is more prevalent in females.^19^ Demographic characteristics can be predictors of fatigue;^20^ in our study population, low socioeconomic level, age, and comorbidities did not significantly influence the presence of fatigue. Most of the affected patients led a sedentary lifestyle; studies suggest that regular physical exercise that has been adapted to the clinical conditions contribute to the reduction in fatigue and improvement in quality of life.^21,22^

Fatigue may be related to objective laboratory data. Univariate analysis has shown that fatigue is associated with changes in the serum parathyroid hormone, iron, urea, calcium, albumin, and hemoglobin levels; a multivariate analysis detected a relationship between fatigue and serum parathyroid hormone.^7^ Resistance to erythropoietin, independent of the degree of anemia and level of transferrin saturation, is associated with factors related to fatigue.^23^ Clinical indicators are objective and reflect a combination of several symptoms; one symptom alone cannot significantly influence serum and biochemical indicators.^7^ In addition, patients undergoing hemodialysis are monitored by nephrologists who encounter frequent clinical changes, which may influence the correlation with symptoms. In this study, there was no statistically significant difference in the laboratory results between the patients with and without fatigue, which may have been due to the immediate treatment of the biochemical changes. The fact that patients with chronic kidney disease have varying symptoms may reflect a multidimensional issue, which has been suggested in previous clinical studies.^24^

Up to 50% of patients undergoing hemodialysis have some degree of depression that impacts the quality of life, decreases the adherence to treatment, and increases the rate of suicide and mortality.^25^ Tryptophan metabolites, the precursors of serotonin and melatonin, are increased in patients undergoing dialysis and may be associated with depression and fatigue.^26^ Considering that there is a causal relationship between fatigue and depression, the finding of increased fatigue in patients undergoing dialysis suggests the need for further investigation and potential diagnosis of depression. Various types of fatigue, such as physical, mental, and emotional, have been described as precursors of depression; furthermore, fatigue has been reported in 22–49% of patients treated with antidepressants and in depression remission.^27^ However, bivariate analyses indicate that depression is causally correlated with an impact on all fatigue types.^28^ Depression was found to be more prevalent in patients with fatigue in our study.

In patients undergoing hemodialysis, sleep disorders predispose them to complications in general health, mental health, and physical capacity and fatigue. The most prevalent etiologies of sleep disorders include psychological factors, such as stress, anxiety, and depression, metabolic changes, pain, dietary restrictions, dyspnea, fatigue, cramps, and hypocapnia secondary to metabolic acidosis.^29^ Sleep alterations affect 40–83% of patients undergoing dialysis,^29–31^ and their association with restless leg syndrome increases the risk of death^30^. The treatment of restless leg syndrome reportedly leads to an improvement in fatigue-related symptoms.^30^ Patients with chronic kidney disease undergoing hemodialysis have increased tryptophan catabolism, an essential amino acid that increases serotonin production in the central nervous system. A subsequent decrease in the serum concentration of tryptophan could be related to changes in the sleep quality and fatigue.^6,26^ A study demonstrated that non-pharmacological treatments that decreased anxiety were associated with a reduction in fatigue and improvement in sleep quality.^31^ Additionally, a meta-analysis showed that performing aerobic exercises during dialysis sessions and acupuncture sessions can improve the sleep quality and decrease the reliance on drugs for the treatment of sleep disorders;^32^ performing aerobic exercises before the dialysis session could also have similar benefits.^33^ Our study established a significant association between sleep disorders and fatigue, suggesting that the treatment of these disorders could decrease the prevalence of fatigue and improve the quality of life.

The quality of life in patients undergoing hemodialysis influences their prognosis and mortality rate. Thus, diagnosing sleep disorders and fatigue is as an integral part of the treatment.^4,34^ Our results were comparable to that of a study where the quality of life was inversely related to fatigue and depression. Additionally, married patients whose treatments were financially supported experienced a better quality of life.^34^ Our study confirmed this inverse relationship, especially in the domains of physical and general health. Physical exercise programs performed during the intradialytic period reportedly have a positive impact on the patient’s quality of life, depression, and fatigue.^35^

Our study had several limitations. Due to the observational nature of this study, we could not infer a cause-and-effect relationship among the observed variables, that is, between the occurrence of fatigue and depression or sleep disorders. Therefore, caution should be exercised when applying these results to patients undergoing hemodialysis in daily practice. Further prospective studies are needed to determine the etiology of fatigue and assess its prognostic role in patients with chronic kidney disease undergoing hemodialysis.

## CONCLUSIONS

Fatigue is common among patients undergoing hemodialysis and is associated with depression and sleep disturbances. Clinicians should proactively investigate signs of fatigue to avoid its impact on the quality of life in patients with end-stage kidney disease.
